# Genetic and Functional Characterization of Toll-Like Receptor Responses in Immunocompetent Patients With CMV Mononucleosis

**DOI:** 10.3389/fcimb.2020.00386

**Published:** 2020-08-07

**Authors:** Giada Frascaroli, Giada Rossini, Virginia Maltoni, Michele Bartoletti, Patrizia Ortolani, Sara Gredmark-Russ, Francesco Gelsomino, Alessandra Moroni, Silvia Silenzi, Gastone Castellani, Vittorio Sambri, Antonio Mastroianni, Wolfram Brune, Stefania Varani

**Affiliations:** ^1^Heinrich Pette Institute, Leibniz Institute for Experimental Virology, Hamburg, Germany; ^2^Unit of Microbiology, St. Orsola-Malpighi University Hospital, Bologna, Italy; ^3^Department of Experimental, Diagnostic and Specialty Medicine, University of Bologna, Bologna, Italy; ^4^Infectious Diseases Unit, Department of Medical and Surgical Science, University of Bologna, Bologna, Italy; ^5^Infectious Disease Unit, Infermi Hospital, Rimini, Italy; ^6^Center for Infectious Medicine, ANA Futura, Department of Medicine Huddinge, Karolinska Institutet, Stockholm, Sweden; ^7^Division of Infectious Diseases, Department of Medicine Huddinge, Karolinska Institutet, Stockholm, Sweden; ^8^Department of Physics and Astronomy and Galvani Center for Biocomplexity, University of Bologna, Bologna, Italy; ^9^Unit of Microbiology, The Romagna Hub Laboratory, Pievesestina, Italy; ^10^Unit of Infectious and Tropical Diseases, St. Annunziata Hospital, Cosenza, Italy; ^11^Unit of Infectious Diseases, G.B. Morgagni-Pierantoni Hospital, Forlì, Italy

**Keywords:** cytomegalovirus, mononucleosis, toll-like receptors, polymorphism, pro-inflammatory cytokines

## Abstract

**Background:** Human cytomegalovirus (CMV) modulates both innate and adaptive immune responses. However, limited data are available on the role of receptors of innate immunity, such as toll-like receptors (TLRs) in contributing to antiviral responses and inflammation.

**Objectives:** The aim of this translational study was to characterize TLR responses in immunocompetent patients with primary and symptomatic CMV infection.

**Study Design:** The study population consisted of 40 patients suffering from CMV mononucleosis and 124 blood donors included as controls. We evaluated the association between TLR2, 3, 4, 7 and 9 gene single nucleotide polymorphism (SNP) and susceptibility to symptomatic CMV infection in immunocompetent adults. Additionally, functional TLR-mediated cytokine responses in supernatants of short-term cultures of whole blood from patients with CMV mononucleosis and blood donors were evaluated.

**Results:** TLR2 and TLR7/8 responses were altered in CMV infected patients as compared to healthy donors and were associated with the release of higher levels of the pro-inflammatory cytokines IL-6 and TNF-α, but not of the anti-inflammatory mediator IL-10. The analysis on the TLR SNPs indicated no difference between patients with CMV infection and the control group.

**Conclusions:** No variation in the TLR2,3,4,7 and 9 genes was associated to the development of symptomatic CMV infection in immunocompetent adults. Nevertheless, TLR-mediated responses in CMV-infected patients appeared to be skewed toward a pro-inflammatory profile, which may contribute to the development of inflammatory symptoms during the CMV mononucleotic syndrome.

## Introduction

Among the ubiquitous herpesviruses, human cytomegalovirus (CMV) encodes the greatest number of genes committed to altering both innate and adaptive immune responses. In the presence of a fully functional immune system, primary CMV infection is usually asymptomatic. However, when the immune system is functionally impaired, such as in immunocompromised patients, CMV disseminates in its host and can cause a broad range of clinical syndromes, including hepatitis, pneumonitis, enterocolitis, encephalitis (Miller-Kittrell and Sparer, 2009). Pathological conditions during CMV infection are caused by direct mechanisms, reflecting viral burden and virus-mediated cell destruction, and by indirect effects that can be observed even in the presence of low levels of CMV replication and that depend on the viral activation of the host's immune and inflammatory responses. *In vitro* and *in vivo* studies indicate that CMV causes a chronic activation of the immune system and a sustained inflammatory response. Pro-inflammatory cytokines produced during CMV infection contribute to tissue damage and CMV may affect immune privileged organs, where immune-mediated pathology can ensue (Clement and Humphreys, 2019). Signs of active viral infection have also been identified in inflammatory lesions of autoimmune diseases, including inflammatory bowel diseases, where an association between CMV infection and exacerbation of inflammation has been found (Sager et al., 2015; Pillet et al., 2016; Romkens et al., 2016). Further, CMV infection generates a chronic pro-inflammatory state; both CMV active and latent infection induce sustained inflammatory responses that are accompanied by a type 1 cytokine signature in transplant patients and in healthy individuals (van de Berg et al., 2010). Evidence also suggests an intriguing role for CMV in persistence of systemic inflammation during recovery from critical illness, likely contributing to worse prognosis (Griffith et al., 2016).

The mechanisms by which CMV triggers inflammation are only partially understood. During infection, CMV is sensed by several classes of pathogen recognition receptors, including toll-like receptors (TLRs) that in turn activate the first line of host defense triggering inflammatory cytokine secretion and, in most cases, type I IFN production. Components of the CMV envelope such as the glycoproteins gB and gH, are recognized by TLR2 and TLR4 expressed on the cell plasma membrane (Compton et al., 2003; Juckem et al., 2008). On the other hand, the CpG rich CMV genomes as well as the viral RNA intermediate species are recognized by the endosomal TLR3, 7 and 9 (Crane et al., 2012). The genes encoding TLRs are extremely polymorphic and several studies have reported the association between single nucleotide polymorphisms (SNPs) in the TLRs and enhanced susceptibility and severity to systemic infections (Schroder and Schumann, 2005; Netea et al., 2012; Sezgin et al., 2019).

The aim of this study was to characterize TLR polymorphism and the functional responses to their cognate ligands in immunocompetent subjects undergoing a primary and symptomatic CMV infection. It is well known that only few immunocompetent individuals experience mononucleosis symptoms due to CMV infection. In this study, we investigated whether CMV mononucleosis was associated with improper TLR functional responses and/or to the presence of SNPs within TLRs selected for their possible functional effects (TLR2 Arg753Gln; TLR3 Pro554Ser; TLR4 Asp299Gly; TLR7 Gln11Leu, and TLR9−1237 T/C).

## Patients and Methods

### Study Population and Blood Collection

The study population consisted of 40 individuals who visited the Units of Infectious Diseases at the St. Orsola-Malpighi University Hospital (Bologna, Italy), the Morgagni Hospital (Forlì, Italy), the Infermi Hospital (Rimini, Italy) and the Karolinska University Hospital (Stockholm, Sweden) between October 2004 and March 2013. A diagnosis of CMV mononucleosis was reached when a compatible clinical and laboratory profile (malaise, protracted fever, increased liver transaminases, and peripheral blood lymphocytosis with atypical lymphocytes) was associated with the following serological findings: presence of CMV-specific IgM with or without detectable IgG against CMV and lack of serological evidence of primary Epstein-Barr virus infection. CMV specific IgM and IgG were detected by Enzygnost (Siemens Healthcare GmbH, Erlangen, Germany) at the St.Orsola Malpighi University Hospital (Bologna, Italy) and at the Karolinska University Hospital (Sweden), while Liaison® CMV IgG and IgM (Diasorin) was employed at the Unit of Microbiology, The Romagna Hub Laboratory (centralized laboratory for Morgagni Hospital and Infermi Hospitals). Quantification of CMV DNA in peripheral blood was retrospectively performed by real-time polymerase chain reaction for the CMV major immediate early region, as described in Varani et al. (2009). Furthermore, anti-EBV IgM and IgG detection was performed by Enzygnost (Siemens) at the St.Orsola Malpighi University Hospital (Bologna, Italy) and at the Karolinska University Hospital (Sweden), while Liaison® EBV IgM, VCA IgG and EBNA IgG (Diasorin) was employed at the Unit of Microbiology, The Romagna Hub Laboratory. As healthy controls, blood samples from 124 Caucasian blood donors (age range 18–65, 89 male) were obtained from the Blood Bank, St.Orsola-Malpighi University Hospital, Bologna, Italy.

The study was conducted in accordance with the Declaration of Helsinki, and the protocol was approved by the Ethics Committee of the St. Orsola Malpighi University Hospital, Bologna, Italy (Ref. nr. 91/2011/U/Tess), Forlì Hospital and Rimini Hospital, Italy (Ref. nr. 4093/F2) and the regional ethical review board in Stockholm, Sweden (Ref. nr. 04-039/3 and 2011/893-32). According to the study protocol, only Caucasian patients were included in the study. Written informed consent was obtained from all patients.

For functional studies, blood samples were collected from 16 CMV-infected patients (within 5 days after diagnosis of CMV mononucleosis) and from 18 blood donors. Six to eight milliliters of peripheral blood were drawn from patients and healthy controls, collected into K-EDTA tubes and processed for the study within 30 h from collection. Whole blood samples were stored at −80°C for immunogenetic analysis.

### Genotyping of TLR Variants

TaqMan SNP genotyping Assay with TaqMan genotyping Master Mix (Thermo Fisher Scientific Foster City, CA, USA) was used to investigate the presence of specific SNPs in TLR genes: TLR2 *Arg753Gln* (rs5743708) (Kijpittayarit et al., 2007); TLR3 *Pro554Ser* (rs121434431) (Zhang et al., 2007); TLR4 *Asp299Gly* (rs4986790) (Cervera et al., 2007); TLR7 *Gln11Leu* (rs179008) (Schott et al., 2008; Oh et al., 2009; Arav-Boger et al., 2012) and TLR9−1237 T/C (rs5743836) (Oliveira et al., 2013). The selection of SNPs was based on their possible functional effects and on the existence of previously demonstrated association with infectious diseases.

### Stimulation of Blood Cells With Specific TLR Ligands

Whole blood samples were diluted 1:1 with RPMI medium prior to distribution in separate 200 μl aliquots into a 96-well flat bottom plate for cell cultures. Diluted blood samples were left unstimulated or stimulated either with *Escherichia coli* 0111:B4 peptidoglycan (PGN EB, TLR2 ligand, 5 μg/mL, InvivoGen, San Diego, CA, USA); poly(A:U) (TLR3 ligand; 20 μg/mL; InvivoGen); ultrapure lipopolysaccharide from *E. coli* (LPS, TLR 4 ligand; 100 μg/mL, Sigma-Aldrich, Schnelldorf, Germany); Imiquimod (TLR7 ligand; 20 μg/mL, InvivoGen); Resiquimod-R848 (TLR 7/8 ligand; 10 μg/mL, InvivoGen), or CpG oligonucleotide type A (CpG ODN2216; TLR9 ligand; 3 μg/ml; Metabion, Martinsried, Germany). After 24 h of incubation at 37°C in 5% CO_2_, culture supernatants were collected by centrifugation and stored at −80°C for cytokine determination.

### Determination of Cytokine Levels in Supernatants

The levels (pg/mL) of IL-6, IL-10, IFN-γ, and TNF-α were measured in blood cell supernatants by employing the Magnetic Luminex Screening Assay Kit (R&D Systems, Bio-Techne GmbH, Wiesbaden-Nordenstadt, Germany) and a Luminex 200™ instrument (Luminex Corporation, Austin, TX, USA). The assay sensitivity was 1.7 pg/mL for IL-6; 1.6 pg/mL for IL-10; 0.40 pg/mL for IFN-γ, and 1.2 pg/mL for TNF-α.

### Statistical Analysis

Due to the non-parametricity of the experimental data, the Mann–Whitney U test was used to evaluate the significance between groups of cytokine levels in healthy donors vs. CMV-positive patients. A *p*-value equal to or <0.05 was considered significant. The association between TLR polymorphisms and CMV infection has been tested by Fisher exact test (in the case of 2 by 2 contingency table) and by Chi-square test (in the case of 2 by 3 tables). Because TLR7 is located on the X chromosome, male and female individuals were also analyzed separately.

## Results

### Patients Characteristics

From October 2004 to March 2013, 40 adults with CMV mononucleosis were admitted to the Infectious Disease Units at the St. Orsola-Malpighi University Hospital (Bologna, Italy), the Morgagni Hospital (Forlì, Italy), the Infermi Hospital (Rimini, Italy) and the Karolinska University Hospital (Stockholm, Sweden). Mononucleosis was spontaneously acquired and not transfusion related in all cases. CMV mononucleosis syndrome was diagnosed on the basis of clinical data and laboratory findings, as described (Frascaroli et al., 2006). The mean age of CMV-infected patients was 39 years (range 24–68); 25 patients were male (62.5%). Low levels of CMV DNA in peripheral blood were observed in 32 out of 32 patients during the acute phase of infection (median value 1320 viral genomes/mL, range 10-73100 genomes/mL). Results of quantitative PCR were not available for 8 patients out of 40.

### TLR Variations in Patients With CMV Mononucleosis

The presence of selected SNPs within TLR2, TLR3, TLR4, TLR7 and TLR9 was analyzed in the 40 patients with CMV mononucleosis and 124 blood donors by TaqMan SNP genotyping. The distribution of the frequencies for the TLR2 rs5743708, TLR3 rs121434431, TLR4 rs4986790, TLR7 rs179008 and TLR9 rs5743836 polymorphisms is summarized in Table 1. The difference in TLR frequency between patients and blood donors was tested with non-parametric test, such as location and Mann-Whitney test. No statistical difference was found, even after grouping blood donors and CMV patients for gender. As a further test, we looked for the association of TLR mutations with CMV infection by using contingency table method, but again no association was found.

**Table 1:** Frequency of TLR genotypes and their association with CMV infection.

**SNP genotype**	**Blood donors *N* (%)**	**CMV +** ***N* (%)**	***p-values***
TLR2 Arg753Gln rs5743708			0.58
GG	123 (99)	40 (100)	
GA	1 (1)	0 (0)	
AA	0 (0)	0 (0)	
TLR3 Pro554Ser rs121434431			0.59
GG	68 (55)	20 (50)	
GA	46 (37)	17 (43)	
AA	10 (8)	3 (8)	
TLR4 Asp299Gly rs4986790			0.94
AA	108 (87)	3*5* (87.5)	
AG	13 (11)	3 (7.5)	
GG	3 (2)	2 (5)	
TLR9−1237 T/C rs5743836			0.60
TT	92 (74)	28 (70)	
TC	31 (25)	12 (30)	
CC	1 (1)	0 (0)	
TLR7 Gln11Leu rs179008			
Female			0.84
AA	22 (63)	9 (60)	
AT	10 (27)	3 (20)	
TT	3 (9)	3 (20)	
Male			0.21
A-	71 (80)	17 (68)	
T-	18 (20)	8 (32)	
Female + Male			0.22
AA, A-	93 (75)	26 (65)	
A/T	10 (8)	3 (8)	
TT, T-	21 (17)	11 (28)	

### CMV Infection Enhances Pro-inflammatory Cytokine Responses Upon TLR Stimulation

**Figure 1:**
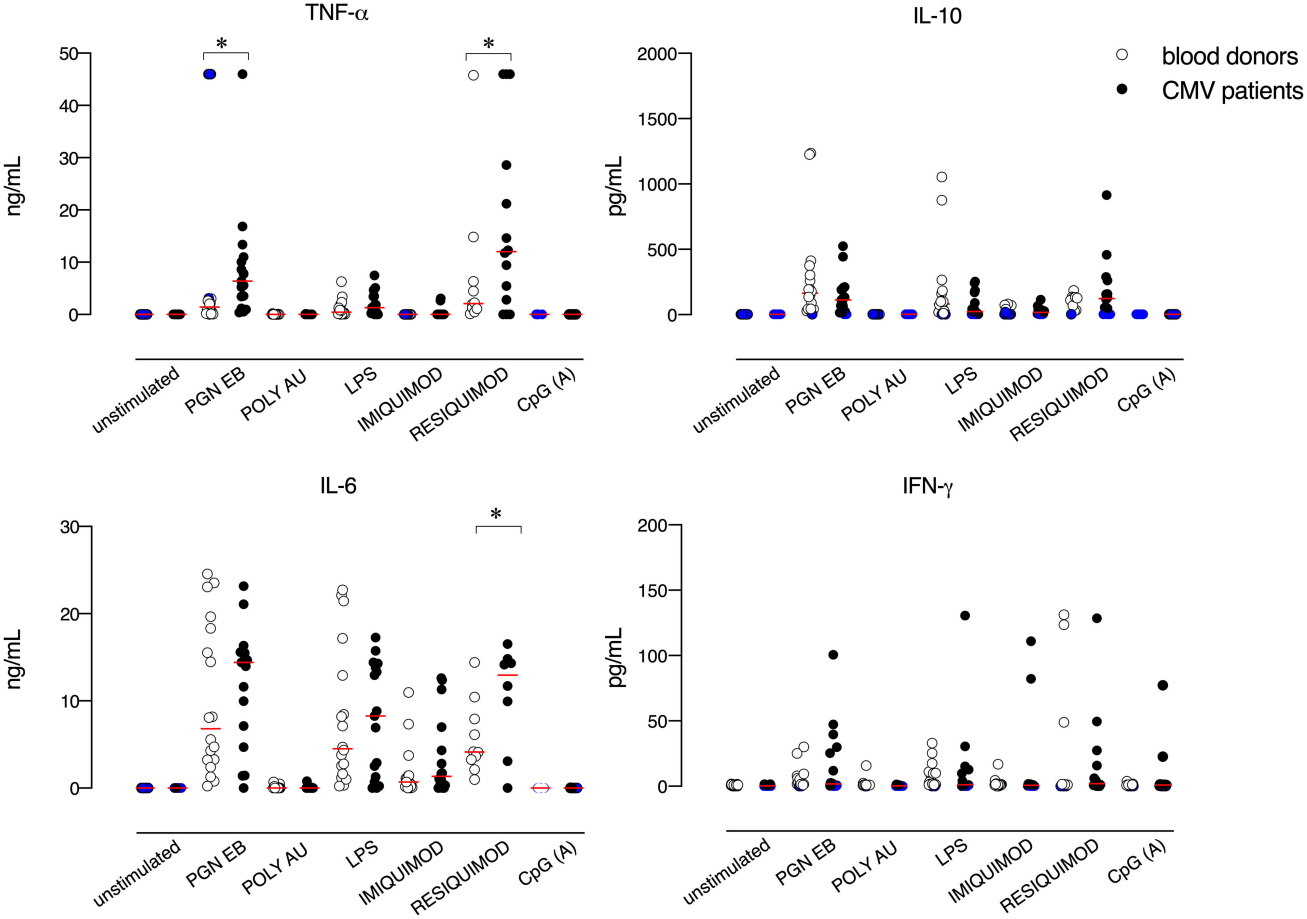
TLR-induced cytokine responses in blood cells from CMV-infected patients and blood donors. Blood cells were left unstimulated or stimulated with PGN EB (TLR2 ligand, 5 μg/mL), poly(A:U) (TLR3 ligand, 20 μg/mL), lipopolysaccharide (LPS, TLR4 ligand, 100 μg/mL), Imiquimod (TLR7 ligand, 20 μg/mL), Resiquimod-R848 (TLR 7/8 ligand, 10 μg/mL) or CpG oligonucleotide type A (CpG ODN2216, TLR9 ligand, 3μg/mL). After 24 h, supernatants were collected and analyzed for cytokine levels. White circles indicate healthy controls and black circles indicate CMV-infected patients. N =16 CMV-infected patients and n = 18 healthy controls were evaluated. Blue circles indicate values below or above the assay's detection limits. Red lines represent mean values. Data were analyzed using the non-parametric Mann–Whitney U test. *p ≤ 0.05.

To examine whether CMV infection impacts TLR-driven host responses, we compared the secretion of pro-inflammatory cytokines from the whole blood of 16 CMV mononucleosis patients and 18 controls stimulated with TLR2, TLR3, TLR4, TLR7, TLR7/TLR8, and TLR9 specific agonists. No difference was observed between the spontaneous release of cytokines by blood cells obtained from CMV-infected patients and blood donors, in the absence of exogenous stimulation (Figure 1). The secretion of TNF-α, IL-6, and IL-10 was induced by TLR2, TLR4, and TLR7/8 agonists, while stimulation with TLR3 and TLR9 agonists, poly A:U and CpG type A respectively, had little or no effect on cytokine release. Overall, only very limited TLR-mediated IFN-γ production was observed, with the highest values seen in response to the TLR7/8 agonist.

As compared to controls, blood cells from patients with CMV mononucleosis secreted higher levels of TNF-α, upon TLR2 stimulation. Furthermore, CMV infected patients exhibited higher levels of IL-6 and TNF-α upon TLR7/8 stimulation as compared to blood donors. No significant difference was observed in terms of TLR-mediated release of the anti-inflammatory cytokine IL-10 between blood cells obtained from CMV infected patients and controls.

## Discussion

CMV is recognized by the host innate immune system by various TLRs (Takeuchi and Akira, 2007). At the cell surface, a direct interaction between the viral glycoproteins and TLR2 and TLR4 has been demonstrated. Upon engagement with their ligands, both TLR2 and TLR4 induce the activation of nuclear factor-kB and the release of pro-inflammatory cytokines (Compton et al., 2003). Beside *in vitro* findings, clinical evidence implicates TLR2 in the pathogenesis of CMV infection, as demonstrated by the fact that liver transplant recipients who carry the homozygous Arg753Gln mutation of *TLR2* have a higher incidence of CMV-related disease (Kijpittayarit et al., 2007). This clinical finding is explained by *in vitro* data showing that cells with the Arg753Gln mutation in *TLR2* fail to identify the viral glycoprotein gB (Brown et al., 2009). Thus, impaired innate viral recognition may hinder the development of a proper antiviral immune response, resulting in symptomatic disease in immunocompromised patients.

Intracellular endosomal TLRs, including TLR3, TLR7, TLR8, and TLR9, detect nucleic acids and are primarily involved in the initiation of innate antiviral responses upon viral detection (Takeuchi and Akira, 2007). Among intracellular TLRs, great attention has been so far posed to the role of TLR3 and TLR9 upon CMV infection. Recent evidence indicates that the mutation that leads to the replacement of the leucine (L) residue in amino acid position 412 by a phenylalanine (F) residue (L412F) in the *TLR3* ectodomain is associated to an increased risk of CMV disease in children and to higher viremia (Studzinska et al., 2017). In plasmacytoid dendritic cells (DCs) – the main producers of type I IFN—CMV induces IFN-α release by engaging the TLR7 and/or TLR9 pathways (Varani et al., 2007). Moreover, upon CMV infection both plasmacytoid DCs and fibroblasts undergo upregulation of TLR9 expression (Varani et al., 2007; Iversen et al., 2009). Finally, an association between the SNP rs5743836, that alters TLR9 promoter activity, and CMV infection has been reported in kidney transplant recipients (Fernández-Ruiz et al., 2015).

We did not observe any association between the presence of the Pro554Ser TLR3 or rs5743836 variation in *TLR9* and the development of symptomatic CMV infection as well as variations in other TLR genes, in our cohort of immunocompetent hosts. Interestingly, CMV infection enhanced the production of pro-inflammatory cytokines in response to TLR stimulation and indeed increased levels of some soluble mediators were observed by stimulation of blood cells obtained from mononucleosis patients with ligands mimicking both bacterial (TLR2 ligands) and viral (TLR2 and TLR7/8 ligands) products. Our findings are in line with *in vitro* data showing that CMV infection of monocyte-derived macrophages promotes the release of TNF-α, IL-6 and IL-8 upon stimulation with TLR2, TLR4, and TLR5 -ligands (Smith et al., 2014), and suggest that CMV infection can contribute to pro-inflammatory cytokine responses by priming the host immune response to react powerfully to unrelated microbial signals.

Previous data indicate a direct dependency of the major immediate early promoter enhancers of CMV on MyD88-dependent TLR-signaling to ensure expression of immediate early genes and to promote viral replication (Kropp et al., 2015). In CMV infected patients, we observed a pro-inflammatory hyper-response to ligation of TLR2 and TLR7/8, all of which are dependent on the adaptor molecule MyD88 (Takeuchi and Akira, 2010), while ligation of TLR3—which is dependent on the TRIF-adaptor molecule—did not lead to cytokine response. In line with our findings, TLR3 has been shown to have no function in the early phases of the cellular response to CMV in human DCs, as demonstrated by experiments in which TLR3 was silenced before CMV infection (Mezger et al., 2009).

Our study has some limitations. First, due to the low frequency of CMV mononucleosis in immunocompetent individuals, a small number of CMV-infected patients was included. To increase the case's cohort, patients were enrolled in four different hospitals over a relatively long period of time. This has led to other limiting factors, including the employment of different serological methods to identify CMV mononucleosis in different hospitals and the lack of possibility to analyze all patient's samples for CMV DNA.

In conclusion, we found that CMV infection promotes inducible inflammatory responses in infected patients by enhancing pro-inflammatory TLR-mediated responses *ex-vivo*. Our findings corroborate multiple evidences demonstrating that CMV infection generates and/or amplifies inflammation (Varani and Landini, 2011) and provide further evidence on the synergy between CMV infection and inflammation.

## Data Availability Statement

The raw data supporting the conclusions of this article will be made available by the authors, without undue reservation, to any qualified researcher.

## Ethics Statement

The study was conducted in accordance with the Declaration of Helsinki, and the protocol was approved by the Ethics Committee of the St. Orsola Malpighi University Hospital, Bologna, Italy (Ref. nr. 91/2011/U/Tess), Forlì Hospital and Rimini Hospital, Italy (Ref. nr. 4093/F2) and the regional ethical review board in Stockholm, Sweden (Ref. nr. 04-039/3 and 2011/893-32). The patients/participants provided their written informed consent to participate in this study.

## Author Contributions

SV designed the study. GF, GR, VM, FG, and SS performed experiments. GC performed statistical analysis. MB, PO, SG-R, AMo, VS, and AMa enrolled patients and collected clinical, virological and serological data on patients at the different hospitals. GF, SV, GR, GC, and WB analyzed the data and wrote the manuscript. All authors contributed in manuscript revision.

## Conflict of Interest

The authors declare that the research was conducted in the absence of any commercial or financial relationships that could be construed as a potential conflict of interest.
